# Women have a poorer very long-term functional outcome after stroke among adults aged 18–50 years: the FUTURE study

**DOI:** 10.1007/s00415-016-8042-2

**Published:** 2016-04-02

**Authors:** Nathalie E. Synhaeve, Renate M. Arntz, Mayte E. van Alebeek, Jeske van Pamelen, Noortje A. M. Maaijwee, Loes C. A. Rutten-Jacobs, Henny C. Schoonderwaldt, Paul L. M. de Kort, Ewoud J. van Dijk, Frank-Erik de Leeuw

**Affiliations:** Department of Neurology, Donders Institute for Brain, Cognition and Behaviour, Radboud University Medical Center, PO Box 9101, 6500 HB Nijmegen, The Netherlands; Department of Neurology, Elisabeth Hospital, Tilburg, The Netherlands; Neurology Unit, Department of Clinical Neurosciences, University of Cambridge, Cambridge, UK

**Keywords:** Stroke in young adults, Prognosis, Functional outcome, Transient ischemic attack

## Abstract

Due to their young age young stroke survivors have to cope with a dramatic impact on their life for the decades to come. We investigated the sex-specific very long-term functional outcome after transient ischemic attack (TIA) and ischemic stroke (IS) in adults aged 18–50 years. This study is part of a cohort study among 619 first-ever young ischemic stroke patients, admitted to our department between January 1, 1980 and November 1, 2010. Functional outcome was assessed during follow-up in 2009–2011 and in 2014–2015 with the modified Rankin Scale (mRS) and instrumental Activities of Daily Living scale (iADL). Risk factors for a poor functional outcome (mRS > 2 and iADL < 8) were calculated by logistic regression analysis. After a mean follow-up of 13.9 (SD 8.2) years, 24.5 % of TIA patients and 44.7 % of IS patients had a poor functional outcome (mRS > 2). When assessing the survivors, 15.2 % of TIA patients and 22.9 % of IS patients had a poor outcome as assessed by iADL. The strongest baseline predictors of poor outcome were female sex (OR 2.7, 95 % CI 1.5–5.0) and baseline NIHSS (OR 1.1, 95 % CI 1.1–1.2 per point increase). In conclusion, 14 years after an ischemic cerebrovascular event in young adults, one out of five IS survivors and one out of ten TIA survivors is still dependent in daily life, with a two to threefold higher risk of a poor outcome in women. This includes a period of life, during which important decisions regarding work and family life have to be made.

## Introduction

The proportion of strokes occurring in young adults has increased over the past decade up to almost 20 % of all strokes [1]. At a young age stroke has a major impact on psychological and social functioning, since in general at this age people are active participants in the community. Consequently, uncertainty about their long-term functional outcome may affect patients’ choices in personal and occupational issues [2].

Recently, we have shown that the functional prognosis of young stroke patients is worse than previously assumed. On average 10 years after an ischemic stroke (IS), 1 out of 8 survivors is dependent in daily life with an even higher proportion dependent with increasing follow-up duration [3]. Also, we found that a substantial proportion of the patients deteriorated from their modified Rankin Scale (mRS) immediately after stroke compared with their mRS at follow-up.

As patients, their caregivers and health professionals rank prognosis after stroke among their top 10 priorities with regards to life after stroke [4], we wanted to investigate whether post-stroke recovery is still possible, even after 10 years of follow-up or longer. As women are overrepresented among the younger (<30 years) stroke survivors [5] and prognosis among female stroke victims in general is known to be poorer [6] we explicitly wanted to investigate the sex-specific very long-term prognosis. In addition, we aimed to evaluate which determinants predicted the risk of a poor very long-term functional outcome and the changes over time therein.

## Methods

This study is part of the “Follow-Up of Transient ischemic attack and stroke patients and Unelucidated Risk factor Evaluation”-study (FUTURE study). The design of the FUTURE study has been described previously [7]. In short, the FUTURE study is a prospective cohort study that aims to investigate causes and consequences of young stroke. The Medical Review Ethics Committee region Arnhem-Nijmegen approved the study and we received consent forms from all participants in the study.

## Patients

The FUTURE study comprised all consecutive patients with a TIA, IS or intracerebral hemorrhage (ICH), between ages 18 and 50 years, admitted to the Radboud University Medical Center Nijmegen, the Netherlands, from January 1, 1980 and November 1, 2010. Only surviving patients with first-ever TIA or IS were included in the present study because of the different pathophysiology underlying ICH. To minimize bias due to changing diagnostic techniques, the World Health Organization definition for TIA and stroke was used [8, 9]. TIA was defined as a rapidly evolving focal neurological deficit, without positive phenomena such as twitches, jerks or myoclonus, with no other than a vascular cause lasting less than 24 h. Stroke was defined as focal neurological deficits persisting for more than 24 h. On basis of radiological findings, stroke was subdivided into ICH and IS.

Exclusion criteria were cerebral venous sinus thrombosis and retinal infarction. For the present study we also excluded patients from whom we could not obtain any of the functional outcome measures at the second follow-up moment in 2014–2015.

Patients were identified through a prospective registry of all consecutive young stroke patients that has been maintained at the department since the 1970’ies [10] with a standardized collection of baseline and clinical characteristics (including demographics, stroke subtype and vascular risk factors). Cardiovascular risk factors at baseline were defined either as a history of a risk factor (mentioned in medical history or the use of medication) or as a risk factor detected during admission or analysis of the stroke as follows: diabetes mellitus as a random blood glucose level greater than 11.1 mmol/L or 2 consecutive fasting venous plasma glucose levels of 7.0 mmol/L or greater [11]; hypertension as systolic blood pressure 135 mmHg or greater and/or diastolic blood pressure 85 mmHg or greater, measured after the first week of the index event; and atrial fibrillation when identified on either an electrocardiogram or during continuous electrocardiographic recording. Smoking was defined as smoking at least one cigarette per day in the year prior to the event.

Furthermore all patients underwent a neurological examination and brain imaging at the time of their index event [7]. The assessment of both the etiology [modified Trial of ORG 10172 in Acute Stroke Treatment (TOAST) classification] [12] and severity (National Institutes of Health Stroke Scale (NIHSS) [13]) at admission was done for all cases retrospectively by a validated approach [14, 15], as these scales did not exist at the time when a substantial proportion of our patients experienced their qualifying event.

### Follow-up

Information on the vital status was available either from the hospital or via the municipality registry [16]. Patients alive were invited for follow-up assessment consisting of questionnaires between November 1, 2009 and January 1, 2012. Subsequently, between August 1, 2014 and January 15, 2015 TIA and IS patients alive were approached by telephone to assess their functional outcome [17] as well as incident events using a standardized interview.

### Functional outcome

Our primary outcome measure was the functional outcome of patients at the second follow-up moment (2014–2015) and the change in functional outcome from the first (2009–2012) to the second follow-up wave. Functional outcome was measured by the mRS [18]. This scale ranges from perfect health without symptoms (0) to death (6). The ability to carry out more complex activities was assessed with the instrumental Activities of Daily Living scale (iADL) [19, 20]. This scale assesses a person’s ability to perform tasks such as using a telephone, doing laundry and handling finances, and leads to a summary score ranging from 0 to 8 (low to high function).

A poor functional outcome was defined as either a mRS > 2 or poor performance on the iADL as a score <8, corresponding with dependency in daily life. Whereas we used a common cut-off value for poor outcome on the mRS (>2) [21–23], there is no such commonly used cut-off value for the iADL. However, loss of independence on any iADL task is usually regarded as an impaired iADL [3, 24]. Therefore we have chosen a cut-off value of <8 to indicate poor functional outcome. A deterioration in functional outcome was regarded as a change from a good functional outcome (mRS ≤ 2/iADL = 8) to a poor functional outcome (mRS > 2/iADL < 8).

### Incident cardiovascular disease

The occurrence of an incident stroke (IS or ICH) or other cardiovascular disease (cardiac disease, including myocardial infarction, coronary artery bypass grafting and percutaneous coronary intervention for coronary artery disease, and peripheral artery disease) was assessed with a standardized, structured questionnaire. Whenever an event was suspected, information was retrieved from the treating physicians and subsequently verified by specialists from the appropriate field, who were blinded to both the index event and functional outcome.

### Statistical analysis

Differences in baseline characteristics between TIA and IS patients were analyzed with Chi-square tests for categorical variables, ANOVA or Kruskal–Wallis tests when appropriate. Descriptive statistics were used to describe the long-term functional outcome (mRS/iADL) and the change in functional outcome between the first and second follow-up moment. Characteristics of patients with a poor functional outcome and those who deteriorated were analysed with Chi-square tests. We repeated this analysis stratified by time of admission (before 1980, 1990–2000 and after 2000), by age at baseline (<35, 35–45 and >45 years) and sex.

By means of logistic regression, we calculated odds ratios (OR’s) with their corresponding 95 % confidence intervals for the risk of poor outcome as assessed by the mRS and the iADL. Covariates in the model were type of index event (IS or TIA), sex, age at event, NIHSS at admission, duration of follow-up, incident strokes and incident cardiovascular disease. Two-sided *p* values of less than 0.05 were considered to indicate statistical significance. The statistical analysis was done using SPSS 20 for Windows.

## Results

**Fig. 1:**
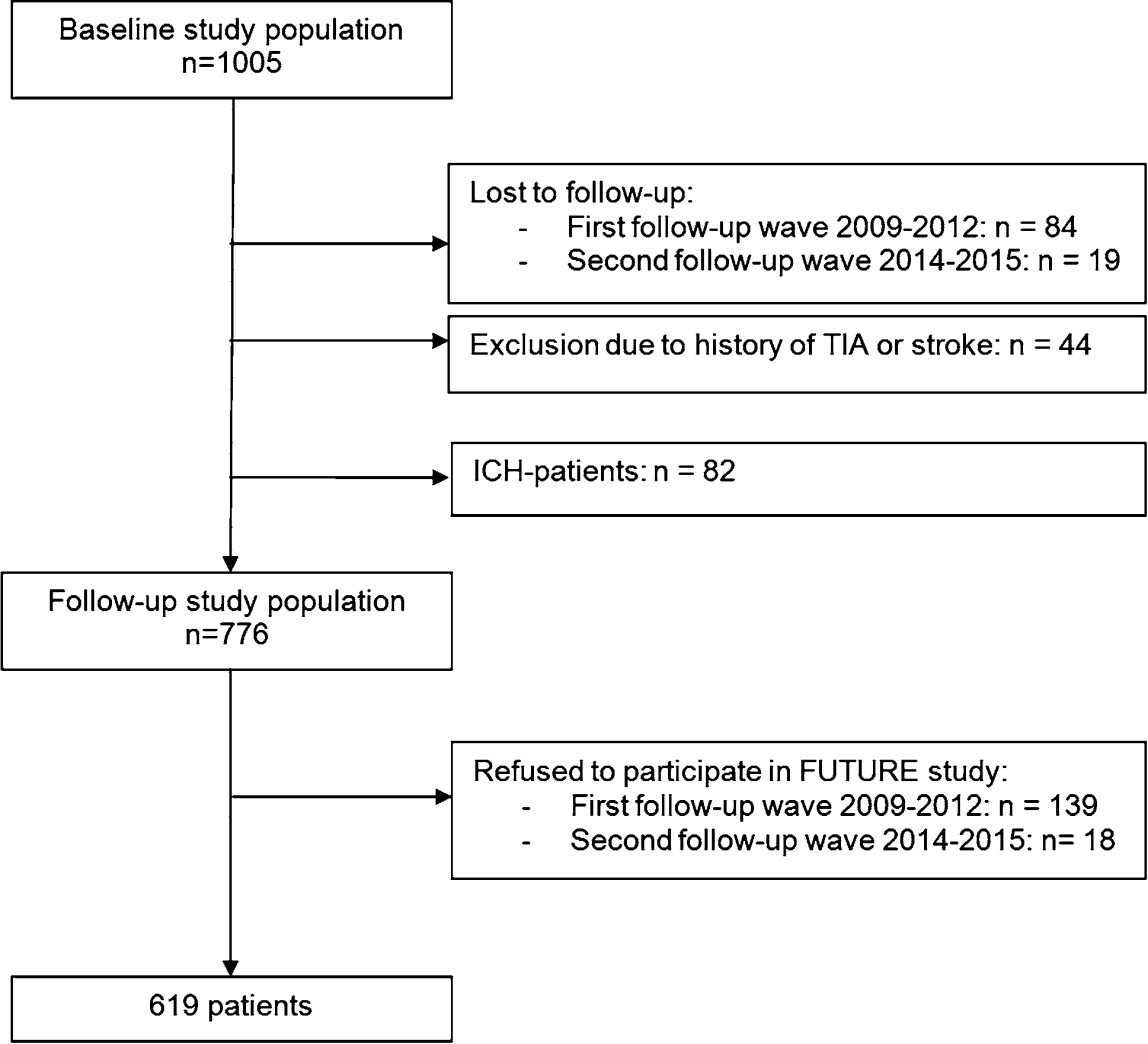
Flow chart of the study population

For the present study, 619 patients with a first-ever TIA and IS were included in the analysis (Fig. 1). Compared with participants, patients who were lost to follow-up more often had an IS (82.6 vs 68.3 %, *p* = 0.005), were slightly younger [38.6 (SD 8.3) vs 40.9 (SD 7.7) years, *p* = 0.010] and less often suffered from hypertension (18.5 vs 36.0 % patients, *p* = 0.001) and diabetes mellitus (2.2 vs 8.4 %, *p* = 0.035). Compared with participants, patients who refused to participate were slightly younger [39.3 (SD 8.0) vs 40.9 (SD 7.7) years, *p* = 0.017] and less often suffered from hypertension (26.1 vs 36.0 %, *p* = 0.019); no differences in any of the other baseline characteristics were found. The baseline characteristics are presented in Table 1. During a mean follow-up of 13.9 (SD 8.5) years 90 patients (14.5 %) suffered from an incident stroke, of which 84 patients (93.3 %) had an IS and 6 (6.7 %) an ICH.

**Table 1:** Baseline characteristics stratified by stroke subtype

	Total	TIA	IS	*p* value
*n* (% of total)	619 (100)	196 (31.7)	423 (68.3)	
Mean age at event, years (SD)	40.9 (7.7)	40.7 (8.0)	40.9 (7.6)	0.903
Men, *n* (%)	296 (47.8)	88 (44.9)	208 (49.2)	0.366
Median NIHSS at admission (IQR)^a^	3 (1–8)	0 (0–1)	5 (2–10)	<0.001
Mean follow-up, years (SD)	13.9 (8.5)	12.3 (8.3)	14.7 (8.5)	0.002
Cardiovascular risk factors, *n* (%)				
Hypertension	223 (36.0)	68 (34.7)	155 (36.6)	0.704
Diabetes mellitus	52 (8.4)	12 (6.1)	40 (6.5)	1 (2.5)
Atrial fibrillation	12 (1.9)	3 (1.5)	9 (2.1)	n/a
Recent smoking^a^	306 (52.0)	78 (40.6)	228 (57.4)	<0.001
TOAST classification, *n* (%)				
Large artery	155 (25.0)	43 (21.9)	112 (26.5)	0.266
Cardio-embolism	81 (13.1)	26 (13.3)	55 (13.0)	1.000
Lacunar	62 (10.0)	11 (5.6)	51 (12.1)	0.019
Other determined	89 (14.4)	21 (10.7)	68 (16.1)	0.100
Multiple	17 (2.7)	2 (1.0)	15 (3.5)	0.127
Undetermined	215 (34.7)	93 (47.4)	122 (28.8)	<0.001

*NIHSS* National Institutes of Health Stroke Scale, *IQR* interquartile range, *TOAST classification* modified Trial of ORG 10172 in Acute Stroke Treatment (TOAST) classification

^a^In 0.5 % of the patients NIHSS at admission and in 4.8 % smoking status was missing

### Functional outcome

After a mean follow-up of 13.9 (SD 8.5) years, a poor functional outcome on the mRS was present in 48 patients (24.5 %) with a TIA and 189 patients (44.7 %) with an IS, of whom 32 TIA patients and 135 IS patients had died at follow-up (mRS = 6). There was a significant difference in the proportion of patients with poor outcome between TIA patients and IS patients (*p* < 0.001). 118 Men (39.9 %) and 119 (36.8 %) women had a poor functional outcome on the mRS (*p* = 0.490), although women were significantly younger (39.7 vs 42.0 years, *p* = 0.001) and had a lower NIHSS (median 2 vs 4, *p* = 0.003) than men.

At follow-up 25 patients (15.2 %) with TIA and 66 patients (22.9 %) with IS had a poor functional outcome on the iADL. This did not significantly differ between TIA and IS patients (*p* = 0.067). 34 Men (16.9 %) and 57 (22.7 %) women had a poor functional outcome on the iADL (*p* = 0.159).

### Transition in functional outcome

**Fig. 2:**
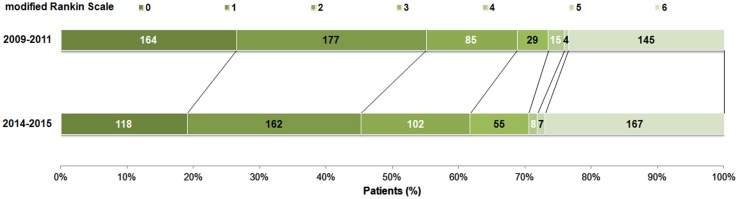
Change of modified Rankin scale scores from first to second follow-up wave (*n* = 619). The *upper bar* represents the mRS data obtained during the first follow-up (2009–2011). The *lower bar* represents data obtained during the second follow-up (2014–2015)

49 patients (11.5 %) deteriorated from good to poor functional outcome between the first (2009–2011) and second (2014–2015) follow-up, of whom 18 had died (Fig. 2). In contrast, only five patients (1.3 %) improved from poor to good functional outcome. Patients with deterioration did not significantly differ in age at index event nor in the decade in which their index event took place compared to patients without deterioration. 12 Of these patients (24.5 %) had suffered from a recurrent stroke compared to 78 patients (13.7 %) of the patients without deterioration (*p* = 0.065). The number of these patients who suffered from another incident vascular event was similar to patients without deterioration (16.3 vs 14.4 %, *p* = 0.874). Women more often deteriorated in functional outcome (as measured with the mRS) than men (10.5 vs 5.1 %, *p* = 0.018).

36 patients (10.3 %) who had an iADL of 8 at the first follow-up moment (on average 5 years ago) now declined to an iADL < 8. Seven patients (15.6 %) with an iADL < 8 at the first follow-up moment improved to a good functional outcome on the iADL. Women more often deteriorated with regards to their functional outcome (iADL) (11.2 % in women vs 6.7 % in men, *p* = 0.171), but this was not significant. No significant differences in the proportion of patients who had suffered from an incident event was seen between patients with and without deterioration.

### Risk factors

Table 2 shows the risk factors for poor functional outcome in young TIA and IS patients. Multiple regression analysis revealed that a poor functional outcome on the mRS was predicted by female sex (OR 2.7; 95 % CI 1.5–5.0), higher age at stroke onset (OR 1.0; 95 % CI 1.0–1.1 per year), higher NIHSS at admission (OR 1.2; 95 % CI 1.1–1.2 per point increase) and the occurrence of an incident stroke (OR 5.7; 95 % CI 2.9–11.2). A poor functional outcome on the iADL was predicted by female sex (OR 2.0; 95 % CI 1.2–3.4), a higher NIHSS at admission (OR 1.2; 95 % CI 1.1–1.3 per point increase) and the occurrence of an incident stroke (OR 5.0; 95 % CI 2.6–9.6).

**Table 2:** Predictors of a poor functional outcome in young TIA and IS patients

	mRS > 2		iADL < 8	
	OR (95 % CI)	*p* value	OR (95 % CI)	*p* value
Ischemic stroke^a^	0.8 (0.4–1.6)	0.537	0.6 (0.3–1.1)	0.099
Female sex	2.7 (1.5–5.0)	0.001	2.0 (1.2–3.4)	0.013
Age at baseline	1.0 (1.0–1.1)	0.026	1.0 (1.0–1.1)	0.118
NIHSS at admission (per point increase)	1.2 (1.1–1.2)	<0.001	1.2 (1.1–1.3)	<0.001
Incident stroke	5.7 (2.9–11.2)	<0.001	5.0 (2.6–9.6)	<0.001
Incident cardiovascular disease^b^	1.1 (0.5–2.4)	0.863	1.0 (0.5–2.2)	0.984
Duration of follow-up (years)	1.0 (1.0–1.1)	0.212	1.0 (1.0–1.0)	0.432

^a^TIA patients serve as the reference group

^b^Incident cardiovascular disease: incident cardiac disease and/or peripheral artery disease

The proportion of patients with a poor functional outcome was 61.6, 47.0 and 23.7 %, depending on the decade of their index event (before 1990, between 1990 and 2000 and after 2000; *p* < 0.001 for overall difference). In patients aged <30, 30–40 and >45 years at the time of their index event the proportion with a poor functional outcome was 27.3, 33.1 and 42.7 % (*p* = 0.015 for overall difference). Between these groups there were no differences in outcome as assessed by the iADL.

## Discussion

We showed that on average 14 years after an ischemic stroke in a young adult almost one out of five long-term survivors (18.8 %) was not able to function independently based on the mRS. In surviving TIA patients this is one out of ten patients (9.8 %). About 1 out of 8 (11.5 %) of the surviving patients with a good functional outcome during the first follow up (on average 10 years after the qualifying event) lost independency in daily life or died during the second follow up (on average 14 years after the event). In contrast, almost one out of six (15.6 %) of the very long time survivors improved to a good functional outcome on the iADL in comparison to the first follow-up moment.

The strength of our study was the long follow-up duration, which is the longest follow-up reported in the field of young stroke, and the ability to detect change in prognosis due to repeated follow-up assessments which enabled us to give more accurate information about the very long-term prognosis after stroke at young age. In addition, our study had a prospective and single center design, which allowed us to collect information systematically and to uniformly verify both the index event as well as the outcome, thereby reducing the risk of information bias, although a single center design could limit the generalizability slightly in comparison to multi-center designs. However, the risk of this is low, therefore we consider it likely that our results can be generalized to most of the young stroke population, especially those who are already months to years past their initial stroke.

However, there are also some limitations. Selection bias may have occurred due to selective loss to follow-up. Both patients lost to follow-up and refusers suffered substantially more often from hypertension at baseline. Therefore non-participants possibly were at a higher risk of incident events and thus an increased risk of a poor functional outcome. Our findings therefore most likely represent an underestimation of the true long-term functional prognosis after stroke in young adults. In addition, possibly not all deficits reported in either of the questionnaires were caused by direct effects of the index stroke, since comorbidity can also lead to the inability to function independently. However, irrespective of the disease underlying the loss of independence, it is remarkable to note the large proportion of still young patients that require help in daily life, on average 14 years after stroke. Finally, during the long inclusion window there have been considerable changes in stroke care, possible resulting in changes in prognosis after stroke at young age as well. We did show a worse prognosis in patients who were admitted with their index event longer ago, however, it cannot be concluded that this is (entirely) due to these changes in care, since these patients also have been at risk to develop recurrent events and comorbidities for a longer period of time.

In previous studies, poor functional outcome in young IS patients ranged from 3 to 7 % after mean follow-up duration between 4 and 12 years [21, 25, 26], better than in our study. This could partly be explained by inclusion of older patients (up to 50 instead of 45 years of age) in our study. Furthermore, in these previous studies functional outcome was measured only by either the mRS or the Glasgow Outcome Scale (GOS), which are commonly used, but are rather global scales for outcome. We intentionally used an additional, complimentary method to determine functional outcome. The iADL (only for survivors) takes into account the ability to perform very specific tasks relevant for living independently, whereas the mRS is a functional outcome measure that is very much dependent on motor performance. These tasks not only rely upon physical health, but also require cognitive function at some level [27]. Consequently the use of these cut-offs of these different scales will result in different groups.

‘Statistics Netherlands’ published data on disabilities in the general Dutch population [28]. Unfortunately the iADL scale is only reported in patients over 55 years of age. However, they do provide information on disability on an OESO-scale, which gives quite a similar impression of disability as the iADL scale. Items on this OESO-scale are walking unaided by another person for 400 m, taking part in a conversation, reading a paper, carrying groceries, picking up an item from the floor. They report that 14.0 % of people between 50 and 55 years (the average age of our patients at follow-up) experience problems on at least one of the items of this scale (in our study 15.2 % of TIA patients and 22.9 % of IS patients experienced problems in iADL). Younger people experience this in ≤7.1 % of the cases. Although this is not a case controlled study, especially IS patients have a much higher (50 %) risk of disability than individuals from the general population.

Finally, we attempted to identify risk factors for long-term poor functional outcome. Interestingly, women had a two to threefold higher risk of a poor outcome than men. This sex-difference in long-term functional outcome after stroke has previously been described in the elderly stroke population [2, 6, 29]. It is assumed that the sex-differences could be (partially) explained by a more advanced age, poorer prestroke functionality, more comorbidities, a tendency to suffer from more severe strokes and less social support in women in comparison to male stroke patients [2, 6, 29]. In our study we observed the increased risk of poor outcome in women despite adjusting for stroke severity, age at initial stroke and the presence of incident events, implying that other underlying mechanisms are involved. There is ongoing research to identify biological mechanisms clarifying the sex-differences in stroke patients, which mainly focuses on the role of sex steroid hormones [29].

Providing patients and relatives with information is an important aspect of clinical practice. Therefore, it is important to realise that female sex and the initial stroke severity are the strongest baseline determinants of poor long-term functional outcome. In addition, higher age at the time of the index event is associated with an increased risk of poor functional outcome. These parameters are objective, easy to assess and readily available in the acute phase. Patients and their caregivers should be informed about the long-term prognosis to give them the opportunity to make educated choices about career moves and family planning.

In conclusion, the very long-term functional prognosis after young stroke is worse than previously assumed. Up to one out of five young ischemic stroke survivors is not able to live independently almost 14 years after stroke. In addition, very long-term poor functional prognosis was seen in almost one out of ten young TIA survivors. This is important information for patients, their caregivers and for health professionals when counselling their patients on the course of the disease. In addition, the continuous decline in functional outcome long after the initial stroke seen in our population suggests that long-term care and follow-up is needed for young stroke patients. Further research is needed to clarify the etiology and to identify explanations of the observed sex differences of the ongoing decline in young stroke patients and to assess whether additional preventive strategies could decrease the proportion of patients with late deterioration.
